# Tumor biology and cancer therapy – an evolving relationship

**DOI:** 10.1186/1478-811X-7-19

**Published:** 2009-08-13

**Authors:** Thomas Seufferlein, Johann Ahn, Denis Krndija, Ulrike Lother, Guido Adler, Götz von Wichert

**Affiliations:** 1 Department of Internal Medicine I, University of Ulm, Albert Einstein Allee 23, 89081 Ulm, Germany; 2 Department of Internal Medicine I, University of Halle, Ernst Grube Strasse 40, 06120 Halle, Germany

## Abstract

The aim of palliative chemotherapy is to increase survival whilst maintaining maximum quality of life for the individual concerned. Although we are still continuing to explore the optimum use of traditional chemotherapy agents, the introduction of targeted therapies has significantly broadened the therapeutic options. Interestingly, the results from current trials put the underlying biological concept often into a new, less favorable perspective. Recent data suggested that altered pathways underlie cancer, and not just altered genes. Thus, an effective therapeutic agent will sometimes have to target downstream parts of a signaling pathway or physiological effects rather than individual genes. In addition, over the past few years increasing evidence has suggested that solid tumors represent a very heterogeneous group of cells with different susceptibility to cancer therapy. Thus, since therapeutic concepts and pathophysiological understanding are continuously evolving a combination of current concepts in tumor therapy and tumor biology is needed. This review aims to present current problems of cancer therapy by highlighting exemplary results from recent clinical trials with colorectal and pancreatic cancer patients and to discuss the current understanding of the underlying reasons.

## Introduction

The aim of palliative chemotherapy is to increase survival whilst maintaining maximum quality of life for the individual concerned. The survival advantage offered by palliative chemotherapy for metastatic cancer has increased incrementally with the addition of each newly licensed therapeutic agent [1]. Still, a number of cancer entities escape these efforts [2]. More recently, advances in the field have led to the introduction of targeted therapies, whose benefits are documented in clinical trials and are acknowledged in their approval and licensing [3-5]. Whilst we are still continuing to explore the optimum use of the more traditional chemotherapy agents, with respect to both quantity and quality of life, these novel agents are trying to find their optimum place in the therapeutic armamentarium. It is evident that a continuing add-one-in policy is likely to be detrimental to both patient and budget. Defining the positioning and duration of these combination therapies has become the subject of much debate and numerous current clinical trials. However, predictions of efficacy based on biological in vitro studies have led to several disappointments lately. At the same time, the understanding of tumor biology is continuously evolving as well. Therefore it is urgently needed to combine results from current trials with current concepts of tumor-biology in order to understand why therapeutic concepts might fail and to possibly help to design future trials more efficiently. This review aims to present and combine current concepts and pitfalls of tumor therapy and tumor biology. We will use exemplary data from current trials mainly from colorectal and pancreatic cancer in order to highlight current problems in tumor therapy. This review does not attempt to cover all aspects related to palliative chemotherapy or all aspects of targeted therapy.

## Signal transduction networks and the response to cancer therapy

An underlying principle of anti-cancer combination chemotherapy is that drugs, that function through separate cytotoxic mechanisms and have different dose-limiting adverse effects, can be administered together at full doses, with a resulting superior outcome [1,2,6]. Parenterally and orally administered fluoropyrimidines have been given with irinotecan in regimens known as FOLFIRI (folinic acid, fluorouracil, and irinotecan) [7] or CapIri (capecitabine and irinotecan) [8] and with oxaliplatin in combinations known as FOLFOX (folinic acid, fluorouracil, and oxaliplatin) or CapOx (capecitabine and oxaliplatin) [9,10]. The use of these drug combinations in metastatic colorectal cancer has prolonged median survival from the 10-to-12-month range associated with fluoropyrimidine therapy alone to more than 20 months. The development of additional effective forms of chemotherapy for colorectal cancer has been possible by the emergence of drugs directed against signaling molecules that are thought to be important in the proliferation of malignant cells [11-13]. This concept has been termed "targeted therapies". Different strategies are available in order to interfere with tumor-specific signal transduction pathways. First the ligand of a given receptor can be targeted. There, two modes of interference are used. i) an antibody targeting the ligand itself (shown for bevacizumab and the vascular endothelial growth factor (VEGF)) [14,15] ii) a decoy receptor competes with the target-receptor for ligand binding (VEGFtrap) [16] or an antibody binds to the receptor, thereby preventing ligand binding (shown for epithelial growth factor receptor (EGFR) binding of cetuximab, panitumumab, EMD72000, h-R3) [17-19]. Alternatively, if the enzyme is to be targeted directly, small molecule inhibitors can be used to interfere with either the substrate binding or with the access to the ATP binding site (e.g. PTK/ZK, erlotinib, gefitinib) [20-23]. Moreover, interfering RNA or DNA molecules can be given systemically in order to posttranslationally inhibit expression of a given gene [24]. Although there is an increasing number of drugs directed towards an equally growing number of target proteins the most commonly used inhibitors are directed against EGFR derived signals (i.e. cetuximab, panitumumab, erlotinib) or VEGF mediated effects (i.e. bevacizumab). Accordingly the use of drugs interfering with these signaling modules has been tested in several trials and we will focus exemplarily on results obtained in colorectal and pancreatic cancer.

**Figure 1:**
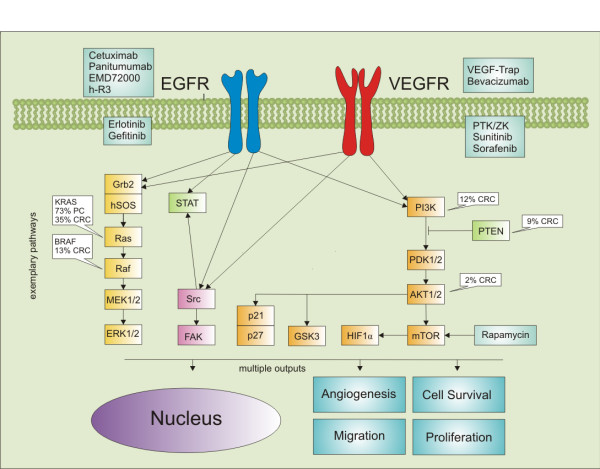
**Exemplary components of the EGFR und VEGR dependent signal transduction pathways**. The compounds/drugs indicated in the green boxes next to the receptors/kinases indicate substances for interference. The white boxes contain the percentages of mutated or altered proteins known to be present in pancreatic cancer (PC) or colorectal cancer (CRC).

Numerous *in vitro *studies have defined multiple components of the EGFR signaling pathway and generated data showing that one or more of these components are commonly activated in colorectal cancer [25,26]. These findings led to preclinical and then clinical trials of EGFR inhibitors. Inhibitors that bind to the EGFR and prevent it from performing its function, namely cell signaling, have been shown to modestly improve progression-free survival, overall survival, and the quality of life among patients with pretreated colorectal cancer [5,27]. Interestingly, the degree of expression of the EGFR in colorectal cancers, as estimated by immunohistochemical analysis, does not appear to predict the efficacy of these antibodies [28,29]. KRAS is a guanosine triphosphate (GTP) hydrolyzing protein that acts as a critical on-off switch in cellular growth and survival pathways [12]. It is a central component of the mitogen-activated protein kinase (MAPK) pathway, which is one of the pathways activated by EGFR signaling [30]. Mutations of KRAS occur in about 40% of colorectal cancers [31]. Recent data suggests that both cetuximab and panitumumab are effective only in the treatment of colorectal tumors with a wild-type KRAS gene; patients with tumors harboring mutations in KRAS are resistant to the two EGFR inhibitors [5,32-35]. In addition, in a recent study mutations were detected in 10% of the patients in the RAS-activated kinase BRAF [36]. Mutations in the RAS and RAF genes are mutually exclusive [37]. None of the patients with tumors exhibiting BRAF mutations responded to an EGFR antibody treatment, wheras none of the responders to the treatment had BRAF mutations [38]. However, not only KRAS and BRAF as immediate downstream targets of the EGFR modulate responsiveness to anti-EGFR therapies. The expression status of the phosphatase and tensin homolog (PTEN) may also affect the clinical response in cetuximab-treated metastatic colorectal cancer patients [39]. In a recent study 27 cetuximab-treated patients were evaluated for drug response. Interestingly, the PTEN protein was normally expressed in 16 patients, and in 10 of them a partial response was achieved. In contrast, no benefit was documented in 11 patients with loss of PTEN activity (P < 0.001) [40]. Similarly levels of other growth factors such as epiregulin and amphiregulin influence the disease control under cetuximab therapy in KRAS wild type tumors [41]. It can be assumed that alterations of more and more proteins (the data mentioned above are only a part of the available data) will be identified that interfere with EGFR signaling and other targeted signaling pathways and that an individualized assessment of eligibility is needed in the future in order to identify patients that benefit from therapeutic efforts (Figure 1).

Rooted in the belief that blocking blood vessel supply starves tumors to death [14], it has become increasingly accepted that blocking tumor angiogenesis as much as possible would provide cancer patients with maximum survival benefit. Given the key importance of VEGF and its receptor VEGFR2 in angiogenesis, hopes were raised that blocking this pathway would eradicate the tumor vasculature and cure cancer. Indeed, the monoclonal anti-VEGF antibody bevacizumab [42,43] and the second-generation multitargeted receptor tyrosine kinase inhibitors (RTKIs) sunitinib [23,44] and sorafenib [3,45] have prolonged the life of numerous cancer patients. Since combining cytotoxic drugs that act through different mechanisms improved outcomes in patients with metastatic colorectal cancer, it seemed logical that further benefit would result from combining bevacizumab with either cetuximab or panitumumab and administering these monoclonal antibodies together with chemotherapy. This assumption was supported by preclinical studies and a phase II clinical trial in 40 patients, that suggested that the bevacizumab-cetuximab combination is associated with acceptable rates of adverse effects [46]. A recent phase III trial conducted in the Netherlands included 732 patients with previously untreated metastatic colorectal cancer who were randomly assigned to receive capecitabine, oxaliplatin, and bevacizumab in cycles administered every 3 weeks or the same regimen supplemented with weekly cetuximab [47]. After a median follow-up of 23 months, the surprising result was that the addition of cetuximab to the combination of capecitabine, oxaliplatin, and bevacizumab significantly decreased the median progression-free survival time from 10.7 months to 9.4 months; the addition of cetuximab was also associated with a trend toward reducing the median overall survival, from 20.3 months to 19.4 months. Tumor tissue was assessed for the status of the KRAS gene in 71% of patients and mutations were found in 40% of the specimens. The addition of cetuximab did not improve the outcome in patients whose tumors contained wild-type KRAS and was also deleterious for those with tumors bearing a mutant KRAS gene. The rate of adverse events was similar in the two treatment cohorts after the exclusion of cetuximab-related adverse cutaneous effects. The negative effect of adding an anti-EGFR antibody to a chemotherapy-bevacizumab combination (CBC) has also been observed by an American study in the Panitumumab Advanced Colorectal Cancer Evaluation (PACCE) trial, in which 823 patients who had not received previous treatment for metastatic colorectal cancer were randomly assigned to receive FOLFOX and bevacizumab, either alone or accompanied by panitumumab [48]. The addition of panitumumab reduced both the median progression-free survival and the median overall survival. At present, there is no obvious explanation for these unanticipated data. The negative effect of combining anti-VEGF and anti-EGFR monoclonal antibodies seems to occur when the two antibodies are administered with chemotherapy regimens that contain either oxaliplatin or irinotecan. The negative effect cannot be attributed to limited treatment intensity due to adverse events, since tolerance of the treatment was indistinguishable in the two groups in the study by Tol et al. [47]. Both Tol et al. and Hecht and colleagues [48] speculate that an unexpected interaction between the two monoclonal antibodies occurred, but no mechanism has yet been identified. These data serve as a reminder that antitumor activity observed in preclinical and also uncontrolled clinical contexts may not be validated when put to the test in randomized trials. Furthermore, the data suggest that combining multiple forms of targeted therapies may not be analogous to combining different types of cytotoxic chemotherapy, presumably because of unrecognized interactions of intracellular signaling pathways. Finally, these results underscore the fundamental importance of subjecting hypotheses to carefully conducted clinical trials. As was observed in these examples, more is not always better.

In contrast to colorectal cancer, there has been no significant improvement of overall survival by chemotherapy in pancreatic cancer patients. However, after a study presented by Burris et al. gemcitabine was established as the gold-standard in the treatment of pancreatic cancer [49]. This study comparing gemcitabine with 5-FU monotherapy showed only a moderate increase in median survival (5.7 months vs. 4.4 months, 1-year survival 18% vs. 2%), but a significant improvement in a new parameter, the so called „clinical benefit response“ (CBR; 23.8% vs. 4.8%). CBR is a construct of a number of parameters including pain-intensity, use of analgetics, functional impairment and change of body-weight. In several confirmatory phase III-trials monotherapy with gemcitabine showed a median survival of 5 to 6.5 months with a 1-year survival of about 11–25%. Many subsequent trials have tested numerous combinations of various cytotoxic agents in the treatment of pancreatic cancer but failed to significantly improve survival [50]. Therefore, the combination of conventional chemotherapy with targeted therapy seemed promising. The EGFR and its ligands are overexpressed in more than 50% of pancreatic cancers. But blocking the EGFR by treatment with the chimeric antibody cetuximab in combination with gemcitabine did not lead to a significant increase in tumor response, progression-free and overall survival [51]. However, a subsequent phase III study was able to show a significant increase in progression-free survival (3.55 vs. 3.75 months) and overall survival (5.91 vs. 6.24 months) by a combination of erlotinib, a small molecule inhibitor of the receptor tyrosine kinase activity of the EGFR, and gemcitabine (HR 0.82, p = 0.038) [52]. Taken the high patient numbers into account (> 280 patients per arm) these small differences were enough to reach statistical significance. Interestingly, a sub-group analysis was able to show, in good agreement with the data obtained in colorectal cancer, that patients developing a skin reaction (rash) ≥ grade II had an improved median survival under combination therapy compared to patients with no skin reaction (10.5 vs. 5.3 months). Another interesting aspect was the improved efficacy in patients with impaired performance status (ECOG 2). However, from a clinical point of view it is not as clear how relevant and meaningful a difference of about 2 weeks in overall-survival, and about 8 days in progression-free survival really is.

As already stated before, in the treatment of colorectal cancer, the combination of a conventional chemotherapy with bevacizumab was able to significantly increase median survival. An analogous phase III study investigating a combination of gemcitabine and bevacizumab in pancreatic cancer (CALGB 80303) was stopped after an interim analysis made an increase in overall survival, the primary endpoint, by the combination therapy unlikely (Kindler et al., Proc ASCO 2007; #4508 (abstract)). The promising data from a non-randomized phase II study could obviously not be reproduced (median survival 8.8 months, 1-year survival 29%) in a larger cohort [53]. In analogy to the colorectal cancer trials, double targeting was analyzed in pancreatic cancer as well. The recent AVITA (BO17706) study was a randomized, double-blind, placebo-controlled phase III study that included 607 patients with metastatic pancreatic cancer. Patients received first-line treatment with either gemcitabine, erlotinib and placebo or gemcitabine, erlotinib and bevacizumab (at 5 mg/kg every two weeks). However, the study did not meet its primary endpoint of improved overall survival (OS) (6.0 vs. 7.1 months), although results showed a slightly improved progression-free survival (3.6 vs. 4.6 months). In addition, further analysis confirmed that skin rash > grade II translated into an increased overall survival [54].

Although it remains speculative why double targeting fails to fulfill the predicted increase in efficacy in several cancer types, recent data provide possible explanations. In a series of elegant papers it was shown that VEGF-targeted drugs inhibit primary tumor growth, yet may shorten survival of mice by promoting tumor invasiveness and metastasis [55,56]. One plausible mechanism for this phenomenon is tumor hypoxia. Unlike normal cells, tumor cells are able to cope with hypoxia more effectively [57]. Apart from metabolic preference for glucose, which allows tumor cells to generate energy under hypoxic conditions, hypoxia-tolerant tumor cell clones are selected, while tumor stem cells in hypoxic niches might escape antiangiogenic treatment as well. Hypoxia thus may select for more malignant metastatic cells, which are less sensitive to antiangiogenic treatment [58]. In support of this concept, treatment of mice with anti-VEGFR2 induces a shift in the glioblastoma tumor phenotype toward enhanced migration and invasion [59]. In addition, tumors devise other vascular supply mechanisms, that are not always inhibited by VEGF-targeted therapy [60], such as the recruitment of angiocompetent bone marrow-derived cells [61] or the co-option of existing vasculature.

How these concepts are in line with current clinical studies is yet to be determined, but further evidence from whole genome analysis of various cancers suggests that effective targeting of individual pathways is probably more difficult than previously suspected [62-64]. In a recent study 20,661 protein coding genes from 24 human pancreatic adenocarcinomas were subjected to genomic analysis to identify changes that occur in the DNA, including SNPs [62]. A typical pancreatic cancer shows 63 genetic alterations, which are mostly point mutations, but many more than 63 genes are involved overall. Of all the genes sequenced, 1327 showed at least 1 mutation, and 148 showed 2 or more mutations in the pancreatic tumor samples. Among the 24 pancreatic cancers studied, 69 gene sets were altered in the majority of tumors. These could be grouped into 12 "core signaling pathways and processes" that each individually affected 67% to 100% of the 24 tumors. Some pathways involved a single altered gene, in others 2 or 3 predominant genes were altered, and other pathways showed alteration of many genes. This in-depth genomic analysis suggested that altered pathways underlie pancreatic cancer and not only altered genes [62]. Thus, for many cancer types, an effective therapy will have to also target downstream parts of these pathways or their physiological effects, such as metabolic functions, expression of cell surface proteins, or changes in the cell cycle, rather than just individual genes.

## Metastasis, cancer stem cell heterogeneity and the response to cancer therapy

Metastasis, the major cause of mortality in patients with cancer, is caused by tumor cells that escape from the primary tumor into the bloodstream and travel through the circulation to distant sites where they develop into secondary tumors. Although these circulating tumor cells (CTCs) provide a link between the primary tumor and metastatic sites, the factors involved in successful survival and eventual formation of metastases are not well understood [65,66]. The number of studies presenting new insights into the mechanisms of colorectal cancer metastases is growing continuously [67-69]. However, it is increasingly appreciated that the number of cells that reach the bloodstream is far greater than the number of metastases [70]. Therefore specific sub-populations of CTCs might be responsible for successful dissemination of cancer cells within an organism.

**Figure 2:**
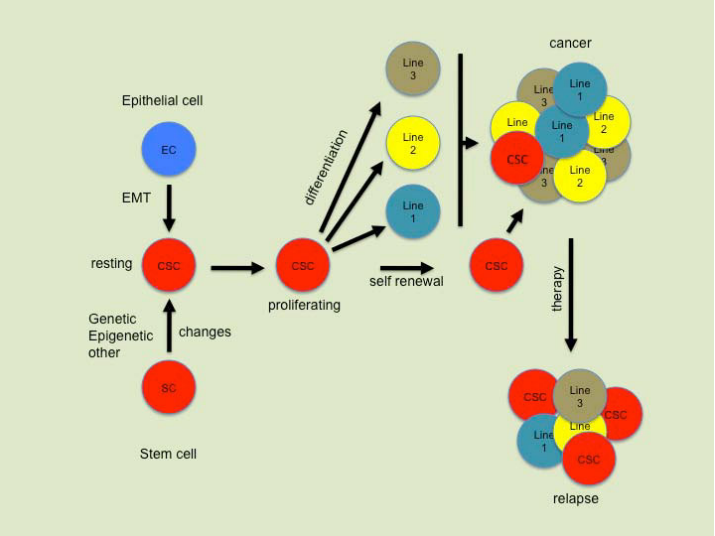
**Possible roles of cancer stem cells (CSC) over the course of the disease**. Cancer stem cells give rise to the different lines of a given tumor. During chemotherapy cancer stem cells are more resistant due to expression of multi-drug-resistance genes.

Over the past few years increasing evidence has suggested that stem cells may play a crucial role in the development and progression of tumors. Distinct populations of cells with stem cell properties may be essential for the development and perpetuation of various human cancers [71-73]. According to the current definition, a cancer stem cell (CSC) represents a cell within a tumor that is able to self-renew, is exclusively tumorigenic, and is capable of producing the heterogeneous lineages of cancer cells that comprise the tumor (Figure 2). In human colorectal cancer a subpopulation of cells with certain surface markers (i.e. CD133+, CD44+ or ESA+ phenotypes) are thought to be uniquely responsible for tumorigenesis and to have the capacity to generate heterogeneous tumors in a xenograft setting. Interestingly, the selection is based on markers that do not have any functional relevance for being a CSC, and the exclusiveness of the CSC population in the ability to initiate cancer as well as its uniqueness in CD133 positivity has been questioned recently [74]. Moreover, recent data suggest that CD133 expression is beyond the rare primitive cells; it rather seems to be a general marker of apical or apicolateral membranes of glandular epithelia [75]. Thus, although it is possible to identify new subsets in cancer cell populations it is increasingly clear that neither the markers for identification nor the underlying mechanisms are sufficiently understood [76,77].

In addition, a specific subpopulation of migrating CSCs has been identified recently and has been attributed with the responsibility for tumor metastasis [72,78]. The stromal-derived factor (SDF)-1(alpha) and the CXC receptor (CXCR)-4 jointly regulate the trafficking of various cell types and play a pivotal role in the migration of hematopoetic cells. A fundamental role of CXCR4 in metastatic spreading has also been suggested for a variety of cancers [79-82]. Interestingly, the distant organ sites most commonly affected by metastases are lymph nodes, lung, liver and bone marrow, all of which exhibit a high content of SDF-1, the natural ligand for CXCR4 [83]. AMD3100, a specific pharmacological inhibitor of the CXCR4 receptor, is currently evaluated in clinical phase I and II trials. The finding that AMD3100 potently inhibits the metastatic activity of unselected murine pancreatic cancer cells [84] as well as other cancer cells [85,86], and the fact that AMD3100 is already evaluated in ongoing clinical trials make it a promising candidate for further experimental studies on CSC-mediated metastasis [87].

Cancer treatment has traditionally been based on the implicit assumption that human cancer populations are homogeneous. A cancer is resilient to treatment because malignant cells survive chemotherapy and radiation or avoid immune surveillance [88]. It has been suggested that the CSC population might be responsible for the resistance of certain tumors to therapy (Figure 2) [89,90]. Gene expression studies revealed a higher expression of DNA mismatch repair genes such as MGMT, as well as genes that inhibited apoptosis in the CD133-expressing cancer stem cells. These included antiapoptotic genes such as FLIP, BCL-2, and BCL-XL [91]. The inhibitor of apoptosis protein family (IAP) genes such as XIAP, cIAP1, cIAP2, NAIP, and survivin were also found at higher expression levels in CD133+ cells. These results were consistent with and support the results of chemoresistance, in which CD133+ cells showed significant resistance to common chemotherapeutic drugs compared with autologous CD133- cells [92]. Interestingly, one way to characterize the CSC population in flow cytometry assays is by Hoechst^® ^dye exclusion [93]. This assay has been commonly used as one of the methods to enrich for cancer stem cells in cancer cell lines and primary tumor cultures [94]. Goodell et al. have demonstrated that the exclusion of Hoechst^® ^dye by CSC is a dynamic process involving the multidrug resistance transporter 1 (MDR1), a member of the ABC transporter transmembrane proteins [95]. MDR1 is certainly not the only transporter present on CSCs since alternative proteins such as the BCRP1 multidrug resistance transporter has been shown to be a molecular determinant of mouse hematopoietic stem cells [96]. Thus, it has to be hypothesized that if non-tumorigenic cells are more susceptible to chemotherapeutic agents, residual tumors after chemotherapy might contain a higher frequency of CSC. Indeed recent data from individual tumor phenotypic analysis and serial transplants suggest that residual tumors after conventional chemotherapy are enriched for cells with a CSC phenotype and have an increased tumorigenic cell frequency [97-99].

These emerging data emphasize the urgent need for either developing targeted therapies against CSCs or modifying current treatment modalities in order to eliminate these cells. Recent studies implicate the Notch signaling pathway as a possible target [100-103]. Using inhibitors of Notch (e.g. OMP-21M18, a humanized monoclonal antibody specific to delta-like 4 (DLL4)), depletion of the specific brain CSC population defined by the CD133 marker or an ability to efflux the Hoechst^® ^dye was shown [104]. Notch was also expressed more highly in the stem cell-like fraction, providing a potential mechanism for their increased sensitivity to inhibition of this pathway. This depletion of the stem cell fraction resulted in a loss of tumor-forming capacity. Apoptotic rates following Notch blockade were increased almost 10-fold in primitive nestin-positive, stem-like cells compared with nestin-negative cells. In addition to that, there is an increasing number of therapeutic strategies specifically targeting the CSC fraction [105]. One further example is parthenolide – a sesquiterpene lactone compound found in Feverfew (Tanacetum parthenium), a traditional medicinal herb that has been used for migraine and rheumatoid arthritis [106]. Parthenolide was shown to have antiproliferative activity on CSCs and is thought to act through inhibition of NF-κB [107].

Other concepts are in the process of evaluation in vitro and in vivo: ARH460-16-2, is a therapeutic antibody targeting CD44 [108], RAV-17 targets another surface molecule of CSCs called B7-H3, GRN163L directly targets the active site of human telomerase and antagonizes telomeric DNA substrates [109], and SL-401 is a chimeric protein comprised of diphtheria toxin fused with the protein IL-3 that appears to be cytotoxic to CSCs is in phase I clinical development [110].

## Conclusion

Taken together, the data presented here emphasize that the development of new treatment strategies is a time consuming and cumbersome process. The results from current trials suggest that new concepts are needed in order to achieve a further increase in survival for these often deleterious diseases. Our understanding of cancer cell biology as well as cancer stem cell biology needs to be deepened in order to identify new targets. A major challenge for the development of new therapeutic agents will be the necessity to discriminate between CSCs and normal stem cells.

## Competing interests

The authors declare that they have no competing interests.

## Authors' contributions

TS Writing and conceptual design. JA Writing and conceptual design. DK Writing and conceptual design. UL Writing and conceptual design. GA Writing and conceptual design. GvW Writing and conceptual design, preparation of the figures. All authors read and approved the final manuscript.

## References

[B1] Meyerhardt JA, Mayer RJ (2005). Systemic therapy for colorectal cancer. N Engl J Med.

[B2] Furukawa T (2008). Molecular targeting therapy for pancreatic cancer: current knowledge and perspectives from bench to bedside. J Gastroenterol.

[B3] Abou-Alfa GK, Schwartz L, Ricci S, Amadori D, Santoro A, Figer A, De Greve J, Douillard JY, Lathia C, Schwartz B, Taylor I, Moscovici M, Saltz LB (2006). Phase II study of sorafenib in patients with advanced hepatocellular carcinoma. J Clin Oncol.

[B4] Hurwitz H (2004). Integrating the anti-VEGF-A humanized monoclonal antibody bevacizumab with chemotherapy in advanced colorectal cancer. Clin Colorectal Cancer.

[B5] Van Cutsem E, Kohne CH, Hitre E, Zaluski J, Chang Chien CR, Makhson A, D'Haens G, Pinter T, Lim R, Bodoky G, Roh JK, Folprecht G, Ruff P, Stroh C, Tejpar S, Schlichting M, Nippgen J, Rougier P (2009). Cetuximab and chemotherapy as initial treatment for metastatic colorectal cancer. N Engl J Med.

[B6] Kabbinavar FF, Hambleton J, Mass RD, Hurwitz HI, Bergsland E, Sarkar S (2005). Combined analysis of efficacy: the addition of bevacizumab to fluorouracil/leucovorin improves survival for patients with metastatic colorectal cancer. J Clin Oncol.

[B7] Folprecht G, Seymour MT, Saltz L, Douillard JY, Hecker H, Stephens RJ, Maughan TS, Van Cutsem E, Rougier P, Mitry E, Schubert U, Kohne CH (2008). Irinotecan/fluorouracil combination in first-line therapy of older and younger patients with metastatic colorectal cancer: combined analysis of 2,691 patients in randomized controlled trials. J Clin Oncol.

[B8] Punt CJ, Koopman M (2008). Capecitabine and irinotecan as first-line treatment of advanced colorectal cancer. J Clin Oncol.

[B9] Cassidy J, Clarke S, Diaz-Rubio E, Scheithauer W, Figer A, Wong R, Koski S, Lichinitser M, Yang TS, Rivera F, Couture F, Sirzen F, Saltz L (2008). Randomized phase III study of capecitabine plus oxaliplatin compared with fluorouracil/folinic acid plus oxaliplatin as first-line therapy for metastatic colorectal cancer. J Clin Oncol.

[B10] Grothey A (2009). A comparison of XELOX with FOLFOX-4 as first-line treatment for metastatic colorectal cancer. Nat Clin Pract Oncol.

[B11] Folkman J, Merler E, Abernathy C, Williams G (1971). Isolation of a tumor factor responsible for angiogenesis. J Exp Med.

[B12] Malumbres M, Barbacid M (2003). RAS oncogenes: the first 30 years. Nat Rev Cancer.

[B13] Messersmith WA, Ahnen DJ (2008). Targeting EGFR in colorectal cancer. N Engl J Med.

[B14] Folkman J (1971). Tumor angiogenesis: therapeutic implications. N Engl J Med.

[B15] Kim KJ, Li B, Winer J, Armanini M, Gillett N, Phillips HS, Ferrara N (1993). Inhibition of vascular endothelial growth factor-induced angiogenesis suppresses tumour growth in vivo. Nature.

[B16] Gomez-Manzano C, Holash J, Fueyo J, Xu J, Conrad CA, Aldape KD, de Groot JF, Bekele BN, Yung WK (2008). VEGF Trap induces antiglioma effect at different stages of disease. Neuro Oncol.

[B17] Tyagi P (2005). Vatalanib (PTK787/ZK 222584) in combination with FOLFOX4 versus FOLFOX4 alone as first-line treatment for colorectal cancer: preliminary results from the CONFIRM-1 trial. Clin Colorectal Cancer.

[B18] Tyagi P (2005). Recent results and ongoing trials with panitumumab (ABX-EGF), a fully human anti-epidermal growth factor receptor antibody, in metastatic colorectal cancer. Clin Colorectal Cancer.

[B19] Schiller JH (2008). Developments in epidermal growth factor receptor-targeting therapy for solid tumors: focus on matuzumab (EMD 72000). Cancer Invest.

[B20] Thomas AL, Morgan B, Horsfield MA, Higginson A, Kay A, Lee L, Masson E, Puccio-Pick M, Laurent D, Steward WP (2005). Phase I study of the safety, tolerability, pharmacokinetics, and pharmacodynamics of PTK787/ZK 222584 administered twice daily in patients with advanced cancer. J Clin Oncol.

[B21] Thomas AL, Trarbach T, Bartel C, Laurent D, Henry A, Poethig M, Wang J, Masson E, Steward W, Vanhoefer U, Wiedenmann B (2007). A phase IB, open-label dose-escalating study of the oral angiogenesis inhibitor PTK787/ZK 222584 (PTK/ZK), in combination with FOLFOX4 chemotherapy in patients with advanced colorectal cancer. Ann Oncol.

[B22] Cascinu S, Berardi R, Salvagni S, Beretta GD, Catalano V, Pucci F, Sobrero A, Tagliaferri P, Labianca R, Scartozzi M, Crocicchio F, Mari E, Ardizzoni A (2008). A combination of gefitinib and FOLFOX-4 as first-line treatment in advanced colorectal cancer patients. A GISCAD multicentre phase II study including a biological analysis of EGFR overexpression, amplification and NF-kB activation. Br J Cancer.

[B23] Motzer RJ, Michaelson MD, Redman BG, Hudes GR, Wilding G, Figlin RA, Ginsberg MS, Kim ST, Baum CM, DePrimo SE, Li JZ, Bello CL, Theuer CP, George DJ, Rini BI (1248). Activity of SU1 a multitargeted inhibitor of vascular endothelial growth factor receptor and platelet-derived growth factor receptor, in patients with metastatic renal cell carcinoma. J Clin Oncol.

[B24] Hau P, Jachimczak P, Schlingensiepen R, Schulmeyer F, Jauch T, Steinbrecher A, Brawanski A, Proescholdt M, Schlaier J, Buchroithner J, Pichler J, Wurm G, Mehdorn M, Strege R, Schuierer G, Villarrubia V, Fellner F, Jansen O, Straube T, Nohria V, Goldbrunner M, Kunst M, Schmaus S, Stauder G, Bogdahn U, Schlingensiepen KH (2007). Inhibition of TGF-beta2 with AP 12009 in recurrent malignant gliomas: from preclinical to phase I/II studies. Oligonucleotides.

[B25] Chu E (2008). Molecular biomarker development for anti-EGFR therapy: moving beyond EGFR expression. Clin Colorectal Cancer.

[B26] Mendelsohn J, Baselga J (2003). Status of epidermal growth factor receptor antagonists in the biology and treatment of cancer. J Clin Oncol.

[B27] Baselga J, Arteaga CL (2005). Critical update and emerging trends in epidermal growth factor receptor targeting in cancer. J Clin Oncol.

[B28] Italiano A, Follana P, Caroli FX, Badetti JL, Benchimol D, Garnier G, Gugenheim J, Haudebourg J, Keslair F, Lesbats G, Lledo G, Roussel JF, Pedeutour F, Francois E (2008). Cetuximab shows activity in colorectal cancer patients with tumors for which FISH analysis does not detect an increase in EGFR gene copy number. Ann Surg Oncol.

[B29] Moroni M, Sartore-Bianchi A, Veronese S, Siena S (2008). EGFR FISH in colorectal cancer: what is the current reality?. Lancet Oncol.

[B30] Vetter IR, Wittinghofer A (2001). The guanine nucleotide-binding switch in three dimensions. Science.

[B31] Bos JL (1989). ras oncogenes in human cancer: a review. Cancer Res.

[B32] Lievre A, Bachet JB, Boige V, Cayre A, Le Corre D, Buc E, Ychou M, Bouche O, Landi B, Louvet C, Andre T, Bibeau F, Diebold MD, Rougier P, Ducreux M, Tomasic G, Emile JF, Penault-Llorca F, Laurent-Puig P (2008). KRAS mutations as an independent prognostic factor in patients with advanced colorectal cancer treated with cetuximab. J Clin Oncol.

[B33] Di Fiore F, Blanchard F, Charbonnier F, Le Pessot F, Lamy A, Galais MP, Bastit L, Killian A, Sesboue R, Tuech JJ, Queuniet AM, Paillot B, Sabourin JC, Michot F, Michel P, Frebourg T (2007). Clinical relevance of KRAS mutation detection in metastatic colorectal cancer treated by Cetuximab plus chemotherapy. Br J Cancer.

[B34] Amado RG, Wolf M, Peeters M, Van Cutsem E, Siena S, Freeman DJ, Juan T, Sikorski R, Suggs S, Radinsky R, Patterson SD, Chang DD (2008). Wild-type KRAS is required for panitumumab efficacy in patients with metastatic colorectal cancer. J Clin Oncol.

[B35] Karapetis CS, Khambata-Ford S, Jonker DJ, O'Callaghan CJ, Tu D, Tebbutt NC, Simes RJ, Chalchal H, Shapiro JD, Robitaille S, Price TJ, Shepherd L, Au HJ, Langer C, Moore MJ, Zalcberg JR (2008). K-ras mutations and benefit from cetuximab in advanced colorectal cancer. N Engl J Med.

[B36] Rajagopalan H, Bardelli A, Lengauer C, Kinzler KW, Vogelstein B, Velculescu VE (2002). Tumorigenesis: RAF/RAS oncogenes and mismatch-repair status. Nature.

[B37] Barault L, Veyrie N, Jooste V, Lecorre D, Chapusot C, Ferraz JM, Lievre A, Cortet M, Bouvier AM, Rat P, Roignot P, Faivre J, Laurent-Puig P, Piard F (2008). Mutations in the RAS-MAPK, PI(3)K (phosphatidylinositol-3-OH kinase) signaling network correlate with poor survival in a population-based series of colon cancers. Int J Cancer.

[B38] Di Nicolantonio F, Martini M, Molinari F, Sartore-Bianchi A, Arena S, Saletti P, De Dosso S, Mazzucchelli L, Frattini M, Siena S, Bardelli A (2008). Wild-type BRAF is required for response to panitumumab or cetuximab in metastatic colorectal cancer. J Clin Oncol.

[B39] Jhawer M, Goel S, Wilson AJ, Montagna C, Ling YH, Byun DS, Nasser S, Arango D, Shin J, Klampfer L, Augenlicht LH, Perez-Soler R, Mariadason JM (2008). PIK3CA mutation/PTEN expression status predicts response of colon cancer cells to the epidermal growth factor receptor inhibitor cetuximab. Cancer Res.

[B40] Frattini M, Saletti P, Romagnani E, Martin V, Molinari F, Ghisletta M, Camponovo A, Etienne LL, Cavalli F, Mazzucchelli L (2007). PTEN loss of expression predicts cetuximab efficacy in metastatic colorectal cancer patients. Br J Cancer.

[B41] Khambata-Ford S, Garrett CR, Meropol NJ, Basik M, Harbison CT, Wu S, Wong TW, Huang X, Takimoto CH, Godwin AK, Tan BR, Krishnamurthi SS, Burris HA, Poplin EA, Hidalgo M, Baselga J, Clark EA, Mauro DJ (2007). Expression of epiregulin and amphiregulin and K-ras mutation status predict disease control in metastatic colorectal cancer patients treated with cetuximab. J Clin Oncol.

[B42] Hurwitz H, Fehrenbacher L, Novotny W, Cartwright T, Hainsworth J, Heim W, Berlin J, Baron A, Griffing S, Holmgren E, Ferrara N, Fyfe G, Rogers B, Ross R, Kabbinavar F (2004). Bevacizumab plus irinotecan, fluorouracil, and leucovorin for metastatic colorectal cancer. N Engl J Med.

[B43] Miller K, Wang M, Gralow J, Dickler M, Cobleigh M, Perez EA, Shenkier T, Cella D, Davidson NE (2007). Paclitaxel plus bevacizumab versus paclitaxel alone for metastatic breast cancer. N Engl J Med.

[B44] Demetri GD, van Oosterom AT, Garrett CR, Blackstein ME, Shah MH, Verweij J, McArthur G, Judson IR, Heinrich MC, Morgan JA, Desai J, Fletcher CD, George S, Bello CL, Huang X, Baum CM, Casali PG (2006). Efficacy and safety of sunitinib in patients with advanced gastrointestinal stromal tumour after failure of imatinib: a randomised controlled trial. Lancet.

[B45] Escudier B, Eisen T, Stadler WM, Szczylik C, Oudard S, Siebels M, Negrier S, Chevreau C, Solska E, Desai AA, Rolland F, Demkow T, Hutson TE, Gore M, Freeman S, Schwartz B, Shan M, Simantov R, Bukowski RM (2007). Sorafenib in advanced clear-cell renal-cell carcinoma. N Engl J Med.

[B46] Saltz LB, Lenz HJ, Kindler HL, Hochster HS, Wadler S, Hoff PM, Kemeny NE, Hollywood EM, Gonen M, Quinones M, Morse M, Chen HX (2007). Randomized phase II trial of cetuximab, bevacizumab, and irinotecan compared with cetuximab and bevacizumab alone in irinotecan-refractory colorectal cancer: the BOND-2 study. J Clin Oncol.

[B47] Tol J, Koopman M, Cats A, Rodenburg CJ, Creemers GJ, Schrama JG, Erdkamp FL, Vos AH, van Groeningen CJ, Sinnige HA, Richel DJ, Voest EE, Dijkstra JR, Vink-Borger ME, Antonini NF, Mol L, van Krieken JH, Dalesio O, Punt CJ (2009). Chemotherapy, bevacizumab, and cetuximab in metastatic colorectal cancer. N Engl J Med.

[B48] Hecht JR, Mitchell E, Chidiac T, Scroggin C, Hagenstad C, Spigel D, Marshall J, Cohn A, McCollum D, Stella P, Deeter R, Shahin S, Amado RG (2009). A randomized phase IIIB trial of chemotherapy, bevacizumab, and panitumumab compared with chemotherapy and bevacizumab alone for metastatic colorectal cancer. J Clin Oncol.

[B49] Burris HA, Moore MJ, Andersen J, Green MR, Rothenberg ML, Modiano MR, Cripps MC, Portenoy RK, Storniolo AM, Tarassoff P, Nelson R, Dorr FA, Stephens CD, Von Hoff DD (1997). Improvements in survival and clinical benefit with gemcitabine as first-line therapy for patients with advanced pancreas cancer: a randomized trial. J Clin Oncol.

[B50] von Wichert G, Seufferlein T, Adler G (2008). Palliative treatment of pancreatic cancer. J Dig Dis.

[B51] Philip PA (2008). Targeted therapies for pancreatic cancer. Gastrointest Cancer Res.

[B52] Moore MJ, Goldstein D, Hamm J, Figer A, Hecht JR, Gallinger S, Au HJ, Murawa P, Walde D, Wolff RA, Campos D, Lim R, Ding K, Clark G, Voskoglou-Nomikos T, Ptasynski M, Parulekar W (2007). Erlotinib plus gemcitabine compared with gemcitabine alone in patients with advanced pancreatic cancer: a phase III trial of the National Cancer Institute of Canada Clinical Trials Group. J Clin Oncol.

[B53] Kindler HL, Friberg G, Singh DA, Locker G, Nattam S, Kozloff M, Taber DA, Karrison T, Dachman A, Stadler WM, Vokes EE (2005). Phase II trial of bevacizumab plus gemcitabine in patients with advanced pancreatic cancer. J Clin Oncol.

[B54] Van Cutsem E, Vervenne WL, Bennouna J, Humblet Y, Gill S, Van Laethem JL, Verslype C, Scheithauer W, Shang A, Cosaert J, Moore MJ (2009). Phase III Trial of Bevacizumab in Combination With Gemcitabine and Erlotinib in Patients With Metastatic Pancreatic Cancer. J Clin Oncol.

[B55] Ebos JM, Lee CR, Cruz-Munoz W, Bjarnason GA, Christensen JG, Kerbel RS (2009). Accelerated metastasis after short-term treatment with a potent inhibitor of tumor angiogenesis. Cancer Cell.

[B56] Paez-Ribes M, Allen E, Hudock J, Takeda T, Okuyama H, Vinals F, Inoue M, Bergers G, Hanahan D, Casanovas O (2009). Antiangiogenic therapy elicits malignant progression of tumors to increased local invasion and distant metastasis. Cancer Cell.

[B57] Brahimi-Horn MC, Chiche J, Pouyssegur J (2007). Hypoxia signalling controls metabolic demand. Curr Opin Cell Biol.

[B58] Yu JL, Rak JW, Coomber BL, Hicklin DJ, Kerbel RS (2002). Effect of p53 status on tumor response to antiangiogenic therapy. Science.

[B59] Kunkel P, Ulbricht U, Bohlen P, Brockmann MA, Fillbrandt R, Stavrou D, Westphal M, Lamszus K (2001). Inhibition of glioma angiogenesis and growth in vivo by systemic treatment with a monoclonal antibody against vascular endothelial growth factor receptor-2. Cancer Res.

[B60] Bergers G, Hanahan D (2008). Modes of resistance to anti-angiogenic therapy. Nat Rev Cancer.

[B61] Grunewald M, Avraham I, Dor Y, Bachar-Lustig E, Itin A, Jung S, Chimenti S, Landsman L, Abramovitch R, Keshet E (2006). VEGF-induced adult neovascularization: recruitment, retention, and role of accessory cells. Cell.

[B62] Jones S, Zhang X, Parsons DW, Lin JC, Leary RJ, Angenendt P, Mankoo P, Carter H, Kamiyama H, Jimeno A, Hong SM, Fu B, Lin MT, Calhoun ES, Kamiyama M, Walter K, Nikolskaya T, Nikolsky Y, Hartigan J, Smith DR, Hidalgo M, Leach SD, Klein AP, Jaffee EM, Goggins M, Maitra A, Iacobuzio-Donahue C, Eshleman JR, Kern SE, Hruban RH, Karchin R, Papadopoulos N, Parmigiani G, Vogelstein B, Velculescu VE, Kinzler KW (2008). Core signaling pathways in human pancreatic cancers revealed by global genomic analyses. Science.

[B63] Parsons DW, Jones S, Zhang X, Lin JC, Leary RJ, Angenendt P, Mankoo P, Carter H, Siu IM, Gallia GL, Olivi A, McLendon R, Rasheed BA, Keir S, Nikolskaya T, Nikolsky Y, Busam DA, Tekleab H, Diaz LA, Hartigan J, Smith DR, Strausberg RL, Marie SK, Shinjo SM, Yan H, Riggins GJ, Bigner DD, Karchin R, Papadopoulos N, Parmigiani G, Vogelstein B, Velculescu VE, Kinzler KW (2008). An integrated genomic analysis of human glioblastoma multiforme. Science.

[B64] Wood LD, Parsons DW, Jones S, Lin J, Sjoblom T, Leary RJ, Shen D, Boca SM, Barber T, Ptak J, Silliman N, Szabo S, Dezso Z, Ustyanksky V, Nikolskaya T, Nikolsky Y, Karchin R, Wilson PA, Kaminker JS, Zhang Z, Croshaw R, Willis J, Dawson D, Shipitsin M, Willson JK, Sukumar S, Polyak K, Park BH, Pethiyagoda CL, Pant PV, Ballinger DG, Sparks AB, Hartigan J, Smith DR, Suh E, Papadopoulos N, Buckhaults P, Markowitz SD, Parmigiani G, Kinzler KW, Velculescu VE, Vogelstein B (2007). The genomic landscapes of human breast and colorectal cancers. Science.

[B65] Goeminne JC, Guillaume T, Symann M (2000). Pitfalls in the detection of disseminated non-hematological tumor cells. Ann Oncol.

[B66] Gupta GP, Massague J (2006). Cancer metastasis: building a framework. Cell.

[B67] Kaplan RN, Psaila B, Lyden D (2006). Bone marrow cells in the 'pre-metastatic niche': within bone and beyond. Cancer Metastasis Rev.

[B68] Saha S, Bardelli A, Buckhaults P, Velculescu VE, Rago C, St Croix B, Romans KE, Choti MA, Lengauer C, Kinzler KW, Vogelstein B (2001). A phosphatase associated with metastasis of colorectal cancer. Science.

[B69] Stein U, Walther W, Arlt F, Schwabe H, Smith J, Fichtner I, Birchmeier W, Schlag PM (2009). MACC1, a newly identified key regulator of HGF-MET signaling, predicts colon cancer metastasis. Nat Med.

[B70] Pantel K, Riethdorf S (2009). Pathology: are circulating tumor cells predictive of overall survival?. Nat Rev Clin Oncol.

[B71] Kucia M, Reca R, Miekus K, Wanzeck J, Wojakowski W, Janowska-Wieczorek A, Ratajczak J, Ratajczak MZ (2005). Trafficking of normal stem cells and metastasis of cancer stem cells involve similar mechanisms: pivotal role of the SDF-1-CXCR4 axis. Stem Cells.

[B72] Hermann PC, Huber SL, Herrler T, Aicher A, Ellwart JW, Guba M, Bruns CJ, Heeschen C (2007). Distinct populations of cancer stem cells determine tumor growth and metastatic activity in human pancreatic cancer. Cell Stem Cell.

[B73] Li C, Heidt DG, Dalerba P, Burant CF, Zhang L, Adsay V, Wicha M, Clarke MF, Simeone DM (2007). Identification of pancreatic cancer stem cells. Cancer Res.

[B74] Shmelkov SV, Butler JM, Hooper AT, Hormigo A, Kushner J, Milde T, St Clair R, Baljevic M, White I, Jin DK, Chadburn A, Murphy AJ, Valenzuela DM, Gale NW, Thurston G, Yancopoulos GD, D'Angelica M, Kemeny N, Lyden D, Rafii S (2008). CD133 expression is not restricted to stem cells, and both CD133+ and CD133- metastatic colon cancer cells initiate tumors. J Clin Invest.

[B75] Karbanova J, Missol-Kolka E, Fonseca AV, Lorra C, Janich P, Hollerova H, Jaszai J, Ehrmann J, Kolar Z, Liebers C, Arl S, Subrtova D, Freund D, Mokry J, Huttner WB, Corbeil D (2008). The stem cell marker CD133 (Prominin-1) is expressed in various human glandular epithelia. J Histochem Cytochem.

[B76] Polyak K, Weinberg RA (2009). Transitions between epithelial and mesenchymal states: acquisition of malignant and stem cell traits. Nat Rev Cancer.

[B77] Mani SA, Guo W, Liao MJ, Eaton EN, Ayyanan A, Zhou AY, Brooks M, Reinhard F, Zhang CC, Shipitsin M, Campbell LL, Polyak K, Brisken C, Yang J, Weinberg RA (2008). The epithelial-mesenchymal transition generates cells with properties of stem cells. Cell.

[B78] Hermann PC, Huber SL, Heeschen C (2008). Metastatic cancer stem cells: a new target for anti-cancer therapy?. Cell Cycle.

[B79] Oonakahara K, Matsuyama W, Higashimoto I, Kawabata M, Arimura K, Osame M (2004). Stromal-derived factor-1alpha/CXCL12-CXCR 4 axis is involved in the dissemination of NSCLC cells into pleural space. Am J Respir Cell Mol Biol.

[B80] Voermans C, van Heese WP, de Jong I, Gerritsen WR, Schoot CE van Der (2002). Migratory behavior of leukemic cells from acute myeloid leukemia patients. Leukemia.

[B81] Wu M, Chen Q, Li D, Li X, Li X, Huang C, Tang Y, Zhou Y, Wang D, Tang K, Cao L, Shen S, Li G (2008). LRRC4 inhibits human glioblastoma cells proliferation, invasion, and proMMP-2 activation by reducing SDF-1 alpha/CXCR4-mediated ERK1/2 and Akt signaling pathways. J Cell Biochem.

[B82] Zhang T, Somasundaram R, Berking C, Caputo L, Van Belle P, Elder D, Czerniecki B, Hotz S, Schuchter L, Spitz FR, Berencsi K, Rani P, Marincola F, Qiu R, Herlyn D (2006). Preferential involvement of CX chemokine receptor 4 and CX chemokine ligand 12 in T-cell migration toward melanoma cells. Cancer Biol Ther.

[B83] Kucia M, Jankowski K, Reca R, Wysoczynski M, Bandura L, Allendorf DJ, Zhang J, Ratajczak J, Ratajczak MZ (2004). CXCR4-SDF-1 signalling, locomotion, chemotaxis and adhesion. J Mol Histol.

[B84] Saur D, Seidler B, Schneider G, Algul H, Beck R, Senekowitsch-Schmidtke R, Schwaiger M, Schmid RM (2005). CXCR4 expression increases liver and lung metastasis in a mouse model of pancreatic cancer. Gastroenterology.

[B85] Zhang S, Qi L, Li M, Zhang D, Xu S, Wang N, Sun B (2008). Chemokine CXCL12 and its receptor CXCR4 expression are associated with perineural invasion of prostate cancer. J Exp Clin Cancer Res.

[B86] Li JK, Yu L, Shen Y, Zhou LS, Wang YC, Zhang JH (2008). Inhibition of CXCR4 activity with AMD3100 decreases invasion of human colorectal cancer cells in vitro. World J Gastroenterol.

[B87] Cashen A, Lopez S, Gao F, Calandra G, MacFarland R, Badel K, DiPersio J (2008). A phase II study of plerixafor (AMD3100) plus G-CSF for autologous hematopoietic progenitor cell mobilization in patients with Hodgkin lymphoma. Biol Blood Marrow Transplant.

[B88] Rich JN, Bao S (2007). Chemotherapy and cancer stem cells. Cell Stem Cell.

[B89] Huff CA, Matsui WH, Douglas Smith B, Jones RJ (2006). Strategies to eliminate cancer stem cells: clinical implications. Eur J Cancer.

[B90] Donnenberg VS, Donnenberg AD (2005). Multiple drug resistance in cancer revisited: the cancer stem cell hypothesis. J Clin Pharmacol.

[B91] Liu G, Yuan X, Zeng Z, Tunici P, Ng H, Abdulkadir IR, Lu L, Irvin D, Black KL, Yu JS (2006). Analysis of gene expression and chemoresistance of CD133+ cancer stem cells in glioblastoma. Mol Cancer.

[B92] Ghods AJ, Irvin D, Liu G, Yuan X, Abdulkadir IR, Tunici P, Konda B, Wachsmann-Hogiu S, Black KL, Yu JS (2007). Spheres isolated from 9 L gliosarcoma rat cell line possess chemoresistant and aggressive cancer stem-like cells. Stem Cells.

[B93] Hirschmann-Jax C, Foster AE, Wulf GG, Nuchtern JG, Jax TW, Gobel U, Goodell MA, Brenner MK (2004). A distinct "side population" of cells with high drug efflux capacity in human tumor cells. Proc Natl Acad Sci USA.

[B94] Ho MM, Ng AV, Lam S, Hung JY (2007). Side population in human lung cancer cell lines and tumors is enriched with stem-like cancer cells. Cancer Res.

[B95] Goodell MA, Brose K, Paradis G, Conner AS, Mulligan RC (1996). Isolation and functional properties of murine hematopoietic stem cells that are replicating in vivo. J Exp Med.

[B96] Zhou S, Schuetz JD, Bunting KD, Colapietro AM, Sampath J, Morris JJ, Lagutina I, Grosveld GC, Osawa M, Nakauchi H, Sorrentino BP (2001). The ABC transporter Bcrp1/ABCG2 is expressed in a wide variety of stem cells and is a molecular determinant of the side-population phenotype. Nat Med.

[B97] Dylla SJ, Beviglia L, Park IK, Chartier C, Raval J, Ngan L, Pickell K, Aguilar J, Lazetic S, Smith-Berdan S, Clarke MF, Hoey T, Lewicki J, Gurney AL (2008). Colorectal cancer stem cells are enriched in xenogeneic tumors following chemotherapy. PLoS ONE.

[B98] Francipane MG, Alea MP, Lombardo Y, Todaro M, Medema JP, Stassi G (2008). Crucial role of interleukin-4 in the survival of colon cancer stem cells. Cancer Res.

[B99] Todaro M, Alea MP, Di Stefano AB, Cammareri P, Vermeulen L, Iovino F, Tripodo C, Russo A, Gulotta G, Medema JP, Stassi G (2007). Colon cancer stem cells dictate tumor growth and resist cell death by production of interleukin-4. Cell Stem Cell.

[B100] Murtaugh LC, Stanger BZ, Kwan KM, Melton DA (2003). Notch signaling controls multiple steps of pancreatic differentiation. Proc Natl Acad Sci USA.

[B101] Stanger BZ, Datar R, Murtaugh LC, Melton DA (2005). Direct regulation of intestinal fate by Notch. Proc Natl Acad Sci USA.

[B102] Weijzen S, Rizzo P, Braid M, Vaishnav R, Jonkheer SM, Zlobin A, Osborne BA, Gottipati S, Aster JC, Hahn WC, Rudolf M, Siziopikou K, Kast WM, Miele L (2002). Activation of Notch-1 signaling maintains the neoplastic phenotype in human Ras-transformed cells. Nat Med.

[B103] Miyamoto Y, Maitra A, Ghosh B, Zechner U, Argani P, Iacobuzio-Donahue CA, Sriuranpong V, Iso T, Meszoely IM, Wolfe MS, Hruban RH, Ball DW, Schmid RM, Leach SD (2003). Notch mediates TGF alpha-induced changes in epithelial differentiation during pancreatic tumorigenesis. Cancer Cell.

[B104] Ridgway J, Zhang G, Wu Y, Stawicki S, Liang WC, Chanthery Y, Kowalski J, Watts RJ, Callahan C, Kasman I, Singh M, Chien M, Tan C, Hongo JA, de Sauvage F, Plowman G, Yan M (2006). Inhibition of Dll4 signalling inhibits tumour growth by deregulating angiogenesis. Nature.

[B105] Fan X, Matsui W, Khaki L, Stearns D, Chun J, Li YM, Eberhart CG (2006). Notch pathway inhibition depletes stem-like cells and blocks engraftment in embryonal brain tumors. Cancer Res.

[B106] Knight DW (1995). Feverfew: chemistry and biological activity. Nat Prod Rep.

[B107] Guzman ML, Rossi RM, Neelakantan S, Li X, Corbett CA, Hassane DC, Becker MW, Bennett JM, Sullivan E, Lachowicz JL, Vaughan A, Sweeney CJ, Matthews W, Carroll M, Liesveld JL, Crooks PA, Jordan CT (2007). An orally bioavailable parthenolide analog selectively eradicates acute myelogenous leukemia stem and progenitor cells. Blood.

[B108] Jin L, Hope KJ, Zhai Q, Smadja-Joffe F, Dick JE (2006). Targeting of CD44 eradicates human acute myeloid leukemic stem cells. Nat Med.

[B109] Harley CB (2008). Telomerase and cancer therapeutics. Nat Rev Cancer.

[B110] Feuring-Buske M, Frankel AE, Alexander RL, Gerhard B, Hogge DE (2002). A diphtheria toxin-interleukin 3 fusion protein is cytotoxic to primitive acute myeloid leukemia progenitors but spares normal progenitors. Cancer Res.

